# Spatial Topological Structure Design of Porous Ti–6Al–4V Alloy with Low Modulus and Magnetic Susceptibility

**DOI:** 10.3390/nano13243113

**Published:** 2023-12-11

**Authors:** Qian Li, Qiang Li, Shasha Lu, Deng Pan

**Affiliations:** 1 School of Mechanical Engineering, University of Shanghai for Science & Technology, No. 516 Jungong Road, Shanghai 200093, China; 2 Materials Genome Institute, Shanghai University, No. 99 Shangda Road, Shanghai 200444, China; dpan_mgi@shu.edu.com

**Keywords:** porous structure, compression properties, low modulus, magnetic susceptibility

## Abstract

Ti–6Al–4V alloy is widely used as a biomaterial for hard tissue replacement, but its Young’s modulus is still higher than that of human bone tissue, which may cause a “stress shielding” effect and lead to implant loosening. In addition, metal implants with low magnetic susceptibility are beneficial for obtaining minimal artifacts in magnetic resonance imaging. To reduce Young’s modulus and magnetic susceptibility of Ti–6Al–4V alloy, a series of irregular prismatic porous structure models were designed based on the Voronoi principle, built by changing the irregularity, prism-diameter-to-initial-seed-spacing ratio, and seed number, and studied using finite-element analysis. Porous samples were prepared by selective laser melting and subjected to a compression test and magnetic susceptibility test. The simulation results show that the prism-diameter-to-initial-seed-spacing ratio has the greatest impact on porosity compared with the irregularity and seed number. The simulation-predicted porosity and compression modulus are highly consistent with the measured ones. The irregular prismatic porous Ti–6Al–4V samples exhibit mechanical properties similar to those of human bones and show a magnetic susceptibility of no more than 50% that of compact Ti–6Al–4V. A regulatable irregular prismatic porous structure is feasible for designing porous implants with desirable properties for biomedical applications.

## 1. Introduction

Recently, several studies have focused on the use of biomaterials. Ti and its alloys, especially commercially pure Ti (cp-Ti) and Ti–6Al–4V, are widely used for hard tissue replacement because of their excellent biocompatibility, high corrosion resistance, low density, low elastic modulus, and high specific strength [1,2,3,4]. They are also suitable for advanced 3D printing, especially referring to the applications with topological constraints [5,6]. However, the Young’s modulus of Ti–6Al–4V (approximately 110 GPa) is still higher than that of human bone (less than 30 GPa) [7], which may cause “stress shielding” [8,9]. Since the 1990s, several researchers have attempted to decrease the Young’s moduli of Ti alloys. Many β-Ti alloys have been developed by adding large amounts of nontoxic β-stabilizing elements, such as Nb, Mo, and Ta, because the β phase has the lowest Young’s modulus among the phases in Ti alloys. Some so-called “neutral” elements, such as Zr and Sn, were also added to improve strength [10]. However, the prices and melting points of the β-stabilizing elements are relatively high, which increases the cost and manufacturing difficulty [11]. In other studies, porous structures were applied to Ti alloys to obtain porous Ti alloys, reducing the bulk density [12]. A lower Young’s modulus is obtained because the porous Ti alloy is no longer a dense material. The increase in porosity results in a decrease in Young’s modulus, which could solve problems associated with stress shielding [13]. In addition, a porous structure can promote the transport of body fluids and stimulate bone ingrowth, which helps improve the fixation of implants to the bone [14,15]. Thus, a porous structure has the advantages of a lower Young’s modulus and better bone growth.

Magnetic resonance imaging (MRI) is widely used to assess implants and the surrounding tissue [16]. During MRI examination, a high magnetic field intensity can magnetize the metallic implants, which results in artifacts and signal loss, hinders the imaging of surrounding structures, and finally affects the diagnosis [17]. The artifact in the obtained images is a shadow that is not a true object but a detected problem resulting from the hardware or software of the MRI device. It has been indicated that the existence of artifacts in MRI results may vary from a few pixels out of balance to a significant distortion of most parts of an image, which would affect the appearance of the object and interfere with the true diagnosis of pathological events [18,19]. The causes of artifacts and factors affecting MRI quality have been widely studied [20]. The artifacts are mainly caused by the large difference in magnetic susceptibility between the implant and the human body [21]. The significant difference in susceptibility between metallic implants and the surrounding tissues causes high local magnetic field variations during MRI and alters the linear conditions, which are necessary to reconstruct the image successfully. Although the severity of artifacts caused by metallic implants can be reduced by carefully adjusting the imaging parameters, this cannot fundamentally solve the problem [22]. Fortunately, a Ti skull net produces only slight artifacts in a 1.5-T MRI system, suggesting that mass reduction can be used to reduce MRI artifacts [23]. Moreover, artifacts are smaller in porous implants with reduced metal mass [24,25].

The porous structure has low density, high specific strength, and large specific surface area, which are beneficial for the adhesion and reproduction of cells, growth of bone tissue, and flow of nutrient solutions [26,27]. Compared with a regular porous structure, an irregular porous structure simulates the microstructure and mechanical properties of natural bone tissue and improves the force distribution, which provides greater opportunities for bionic design. Samples with different pore shapes show different porosities and higher porosity is favorable for the ingrowth of new bone tissue [28]. The optimized irregular porous structure imitates the actual trabecular bone structure well and exhibits a simple and controllable structural design, which achieves considerable coordination and controllability between structural characteristics and mechanical properties and meets the complex and diverse requirements of orthopedic implants [29,30,31]. Bari et al. developed a porous structure that mimics the morphology of bone. Compared with the performance of a bone section replaced by traditional metallic implants, the porous structure reduces stress shielding and stress concentration and meets the requirements for bone ingrowth [32].

Modern design methods for porous structures are based on the use of hierarchical structures obtained from the cell reproduction of known geometric units and features. However, these methods produce porous and interconnected structures that do not imitate the configuration of bone structures and have difficulty reconstructing bone performance. To overcome this drawback, Gómez et al. proposed a method for creating biomimetic scaffolds with a trabecular structure similar to natural bone by developing an interactive generative design (GD) process instead of a classical computer-aided design approach. An irregular porous scaffold established by the Voronoi mosaic method simulated the microstructure of a natural bone well [33]. GD is typically used to establish porous models and multilateral pore models can be created using random functions. In the modeling process, a designated entity is considered the modeling unit, a spatial spline curve is considered the growth path, and an irregular porous structure is constructed using the intersection point of the curve [34,35,36].

With the development of computer-aided engineering (CAE), several types of CAE techniques have been developed, including the finite-element method (FEM), boundary element method, and finite-difference method. The FEM, which is an indispensable part of structural mechanics analysis, was first used by Brekelmans to analyze the stress of bone structures and then became a new research method for orthopedic mechanics [37,38,39].

Additive manufacturing (AM) uses a digital model to build objects layer by layer and is a popular manufacturing method widely used in many fields [40,41,42]. The use of AM has grown rapidly in the biomedical field because it meets the requirements of personalized and rapid manufacturing [43,44,45,46]. In the mid-1990s, selective laser melting (SLM), an AM technology, was developed [47]. SLM uses a laser to instantly melt high-melting-point metal powders to produce metallurgical bonds without adding any low-melting-point elements, binders, or other intermediate materials during manufacturing [48]. Metallic parts with complex shapes directly manufactured by SLM exhibit good mechanical properties, high precision, and nearly 100% relative density; thus, they can be used directly. Because considerable free-design and fast response speed are suitable for processing single small batches and complex-shaped parts, SLM has been used to prepare various implants [49,50,51,52].

In this study, a series of prismatic porous structure models with various structural parameters were designed using the Voronoi principle, and the pore characteristics were studied using the FEM. Porous Ti–6Al–4V samples were prepared by SLM, and their compressive properties and magnetic susceptibilities were subsequently measured.

## 2. Materials and Methods

### 2.1. Modeling

**Figure 1:**
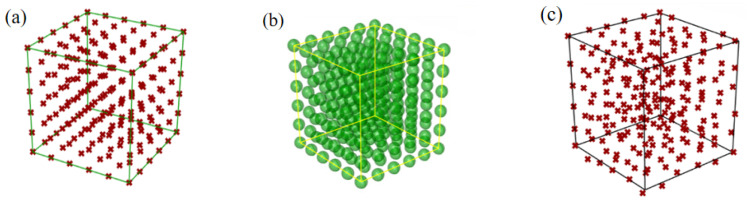

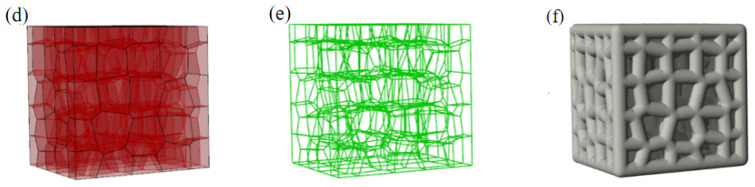
Modeling process of the irregular prismatic porous structure: (**a**) Regular dot matrix; (**b**) Regional sphere with a regular center; (**c**) Irregular dot matrix; (**d**) Voronoi graph; (**e**) Voronoi single-line border; (**f**) Irregular porous structure.

Figure 1 shows the modeling process for the irregular prismatic porous structure. A lattice with a seed number of *n* × *n* × *n* (*n* = 5, 6, 7, 8, 9, 10) was set in a space area of 20 mm × 20 mm × 20 mm (Figure 1a), and the initial seed spacing (*D*) between the two closest dots was 4 mm. A regional sphere was established by centering on the dots (Figure 1b). The diameter of the regional sphere (*d*_s_) was calculated by Equation (1), where the irregularity (*γ*) was set as 0, 0.2, 0.4, 0.6, 0.8, and 1.0 in this study. The seed dots (Figure 1c) for the prismatic porous structure were generated using Equation (2), and the Voronoi tessellation method was performed according to the seed dots (Figure 1d). Single-cell borders were extracted by deleting the polygons (Figure 1e). Finally, the borders were cylindrically processed (Figure 1f) to generate prisms with the prism diameter *(d*_p_*)* fixed by Equation (3), where the *d*_p_-to-*D* ratios (*δ*) were 0.2, 0.4, 0.6, 0.8, and 1.0. Three-dimensional models were established according to the above structural parameters, and the characteristics of the porous structures, including the porosity, specific surface area, and average pore size, were obtained and analyzed.

(1) $$d_{s}=D\times \gamma$$

(2) $$\left\{\begin{array}{l} x_{i}=x+\frac{d_{s}}{2}\times rand\times \sin(\pi \times rand)\times \cos(2\pi \times rand)\\ y_{i}=y+\frac{d_{s}}{2}\times rand\times \sin(\pi \times rand)\times \sin(2\pi \times rand)\\ z_{i}=z+\frac{d_{s}}{2}\times rand\times \cos(\pi \times rand) \end{array}\right.$$

(3) $$d_{p}=D\times \delta$$

where:

*d*_s_—Diameter of regional sphere;
*γ*—Irregularity;
*D*—Initial seed spacing;
*d*_p_—Prism diameter;
*δ*—*d*_p_-to-*D* ratio.

The Gibson–Ashby equation is typically used to evaluate the mechanical properties of porous structures [53,54].

(4) $$\left\{\begin{array}{c} E_{p}=E_{0}\times C_{1}\times {\left(1-\frac{\Phi_{s}}{100}\right)}^{n_{1}}\\ \sigma_{s}=\sigma_{0}\times C_{2}\times {\left(1-\frac{\Phi_{s}}{100}\right)}^{n_{2}} \end{array}\right.$$

where:

*E*_p_—Elastic modulus of porous Ti–6Al–4V;
*E*_0_—Elastic modulus of compact Ti–6Al–4V;
*C*_1_—Constant;
*n*_1_—Constant;
*σ*_s_—Compressive yield strength of porous Ti–6Al–4V;
*σ*_0_—Compressive yield strength of compact Ti–6Al–4V;
*C*_2_—Constant;
*n*_2_—Constant;
*Φ*_s_—Porosity of the sample.

According to the formula, porosity significantly influences the elastic modulus and compressive strength of porous structures, and the modulus and compressive strength of porous structures gradually decrease with increasing porosity. Therefore, in the following experiments, the porosity was considered the most important parameter to study because of its influence on the mechanical properties and magnetic susceptibility. 

### 2.2. Simulations of Compressive Modulus

A 20 mm × 20 mm × 32 mm cubic model was used for the compressive modulus simulation. Prismatic porous structure models with porosities ranging from 30% to 80% were established by changing *δ* and fixing *n* as 6 × 6 × 9 and *γ* as 0.8. The material was Ti–6Al–4V alloy with an elastic modulus of 110 GPa, a density of 4.510 g·cm^−3^, and a Poisson’s ratio of 0.37. A force of 100 N was applied to the top of the model, and the deformation was recorded. The compressive modulus was calculated based on the stress and deformation. 

### 2.3. Preparations and Tests of Porous Samples

Prismatic porous structure models with sizes of 3 × 3 × 3 mm and porosities of 25%, 45%, 65%, and 85% were established by changing *δ* and fixing *n* as 6 × 6 × 6 and *γ* as 0.8. The samples were prepared using an EP-M250 SLM machine with a laser power of 190 W, a scanning speed of 1000 mm·s^−1^, a powder layer thickness of 40 μm, and a spot diameter of 85 μm. The diameter of the SLM used Ti–6Al–4V powders ranging from 15 to 53 μm.

The relative densities of the samples were measured using the Archimedes method. First, the weight of the sample was measured in air (*m*_air_). The sample was then wiped with 75% alcohol to wet its surface and prevent bubbles from escaping from the sample after it entered the water. Subsequently, the error was reduced, and the weight of the sample in water (*m*_water_) was measured. Finally, the porosity of the prepared sample was calculated using the following equation:

(5) $$\Phi_{s}=1-\frac{m_{air}\times \rho_{water}}{\left(m_{air}-m_{water}\right)\times \rho_{material}}\times 100\%$$

where:

*Φ*_s_—Porosity of the sample;
*m*_air_—Weight of the sample in air;
*m*_water_—Weight of the sample in water;
*ρ*_water_—Density of water;
*ρ*_material_—Density of compact Ti–6Al–4V.

The compression testing was performed using an electronic universal testing machine at a speed of 0.2 mm·min^−1^. The magnetization was measured by a PPMS-9 vibrating sample magnetometer under a magnetic field with a magnetic intensity of −3000 to 3000 Oe. 

## 3. Results and Discussion

### 3.1. Structural Characteristics

#### 3.1.1. Porosity

Porosity is the percentage of the pore volume in the model to the total volume and reflects the volume ratio of the solid parts in the model. A larger porosity indicates lower compactness, fewer solids, and larger space inside the model.

**Figure 2:**
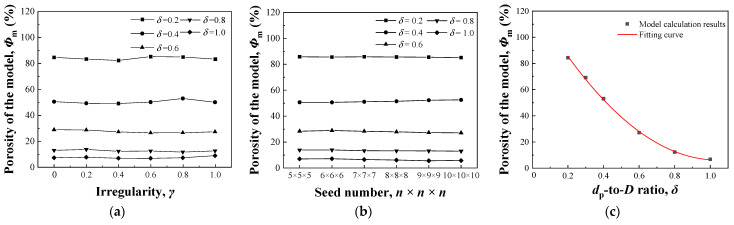
Influence of structural parameters on porosity: (**a**) Irregularity; (**b**) Lattice number; (**c**) *d*_p_-to-*D* ratio.

Figure 2 shows the influence of the structural parameters on the porosity. As shown in Figure 2a, when *δ* is ≥0.6, with the increase in *γ*, the porosity first decreases and then increases, but it is nearly stable with a change range of porosity <0.82%. When *δ* is <0.6, with the increase in *γ*, the porosity decreases first, then increases, and finally decreases, and the overall change range is <1.50%. Generally, the influence of *γ* on porosity is small and can be ignored.

Figure 2b shows the change in porosity with seed number. For the model with a fixed *δ*, the porosity is stable with the increase in seed number, and the overall change range is no more than 1.13%, indicating that the seed number hardly affects the porosity.

Because the influences of irregularity and seed number on the porosity of an irregular prismatic porous structure are minimal, the influences of the two factors on porosity were not considered in the study on the effect of *δ*. Figure 2c shows the change in porosity with *δ* when *γ* is 0.5 and *n* is 6. With the increase in *δ*, the porosity decreases. The relationship between *δ* and porosity is fitted by a quadratic curve with a correlation coefficient of *R* > 0.99. The fitting equation is

(6) $$\Phi_{m}=\frac{112\delta^{2}-233\delta +127.7}{100}$$

where:

*Φ*_m_—Porosity of the model;
*δ*—*d*_p_-to-*D* ratio.

The porosity can be simplified as a single variable function of *δ*, which suggests that the porosity of the prismatic porous structure created by the proposed modeling method has good controllability.

#### 3.1.2. Specific Surface Area

**Figure 3:**
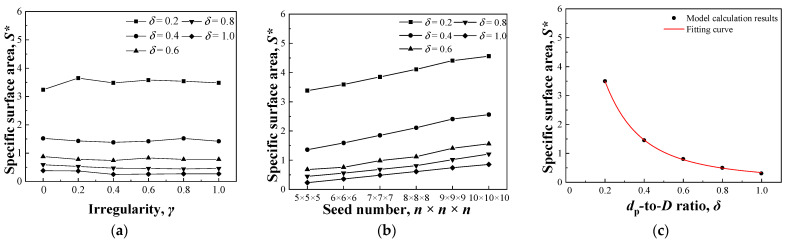
Influence of structural parameters on specific surface area: (**a**) Irregularity; (**b**) Seed number; (**c**) *d*_p_-to-*D* ratio.

As shown in Figure 3a, when *δ* is fixed, the specific surface area minimally changes with the increase in *γ*. Figure 3b shows that the specific surface area increases with increasing seed number. As mentioned above, when *δ* is fixed, the porosity hardly changes, which means that the total solid volume (*V_solid_*) of the model is constant, although the seed number changes. The surface area (*S*_p_) of the prism in the model can be calculated as

(7) $$S_{p}=2\sqrt{\pi hV_{solid}}$$

where:

*V_solid_*—Total solid volume;
h—Total length of the prism.

When the seed number increases, *h* increases; however, *V_solid_* remains unchanged. Therefore, the surface area of the irregular prismatic porous model increases and the specific surface area also increases.

Figure 3c shows the change in specific surface area with *δ* when *γ* is 0.5 and *n* is 6. The relationship between the specific surface area and *δ* is fitted by Equation (8) with the correlation coefficient of *R* > 0.99, suggesting that the specific surface area could be well controlled by adjusting *δ* when *γ* and *n* are fixed.

(8) $$S^{*}=\frac{1}{\left(0.13+2.92\delta^{1.80}\right)}$$

where:

S*—Specific surface area.

The results indicate the specific surface area is not sensitive to irregularity but can be changed by varying seed number and *d*_p_-to-*D* ratio.

#### 3.1.3. Average Pore Size

A linear relationship exists between *δ* and *d*_p_*,* as shown in Equation (3). With the increase in *δ*, *d*_p_ increases, which means the prisms become bigger. Therefore, the prism-enclosed pores become smaller, leading to the decrease in average pore size.

**Figure 4:**
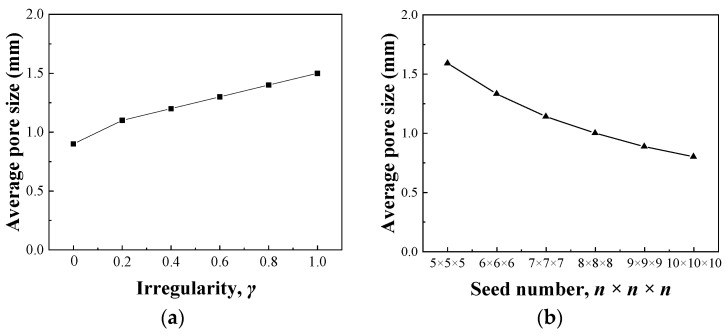
Influence of structural parameters on average pore size: (**a**) Irregularity; (**b**) Seed number.

Figure 4a shows the change in average pore size with *γ* when *δ* is 0.4 and *n* is 6. By computational analysis on the pore size distribution, it is found that when *γ* is 0, the pore sizes are mainly <0.5 mm (small pores) or >2.0 mm (large pores), and medium pores with size of 0.5–2.0 mm are hardly found. This is regarded as a polarization phenomenon of pore size. With increasing *γ*, the polarization phenomenon of pore size gradually disappears, the number of small pores decreases rapidly, and the medium pores become dominant, resulting in the increase in average pore size. This suggests that *γ* can be used to adjust the average pore size.

Figure 4b shows the change in average pore size with seed number when *γ* is 0.5 and *δ* is 0.4. With an increase in seed number, the average pore size gradually decreases. Because the seed number is equal to the pore number, the increased pore number makes the space subdivide into more and smaller pores, decreasing the average pore size.

### 3.2. Simulation Results of Compressive Modulus

**Figure 5:**
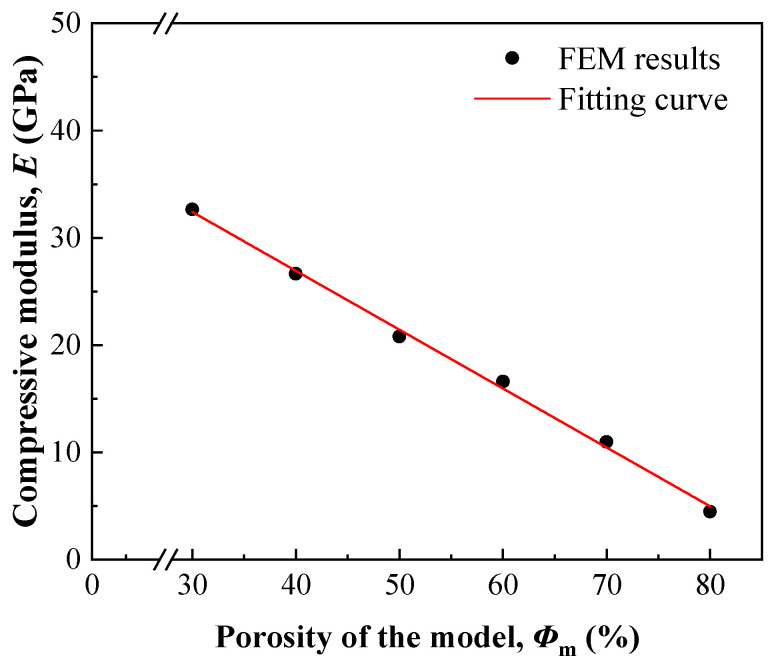
Change in compressive modulus with porosity.

According to Figure 2, *δ* is the most important factor affecting the porosity, which is rarely changed by *γ* and seed number. To study the relationship between compressive modulus and porosity, *δ* was changed with *n* fixed as 6 × 6 × 9 and *γ* as 0.8. As shown in Figure 5, the modulus decreased with increasing porosity. A linear relationship was observed between the modulus and porosity, which was fitted using Equation (9), with a correlation coefficient of *R* > 0.99.

(9) $$E=-53\Phi_{m}+46.9$$

where:

*E*—Modulus;
*Φ*_m_—Porosity of the model.

### 3.3. Test Results of Porous Samples

#### 3.3.1. Porosity

According to Equation (4), the porosity of a porous sample is an important parameter that affects its mechanical properties. Prismatic porous structure models with porosities of 25%, 45%, 65%, and 85% were established by varying *δ* based on Equation (6), and then the porous samples were prepared by SLM using the models. The relative densities of the prepared samples were measured using the Archimedes method, and the porosities were calculated using Equation (5). The values were 21.7%, 43.5%, 63.1%, and 82.2%, respectively. The results suggest that changing *δ* is a feasible way to control the porosity of the sample, and the designed irregular prismatic porous structure is suitable for preparation by SLM.

#### 3.3.2. Compressive Properties

**Figure 6:**
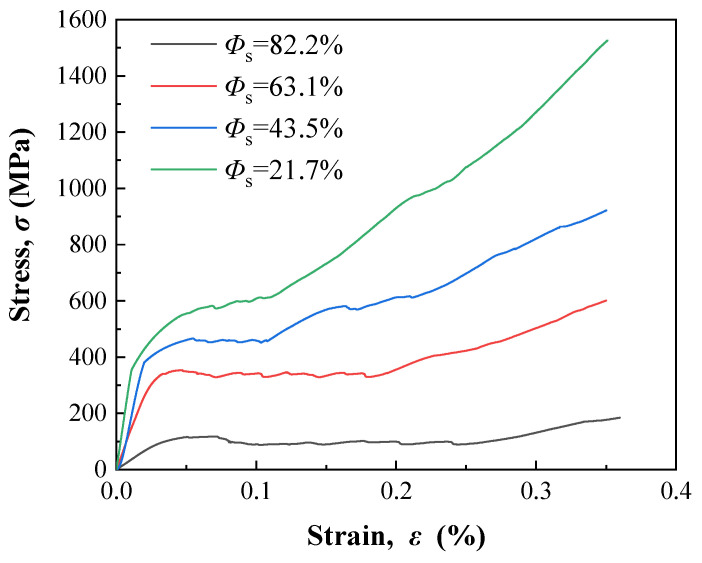
Compressive stress–strain curves of the prepared samples with different porosities.

As shown in Figure 6, all the compressive stress (*σ*)–strain (*ε*) curves of the prepared samples with irregular prismatic porous structures can be divided into three stages, although the samples have different porosities and show different compressive properties. The first stage corresponds to the elastic deformation of the sample. It is the initial stage of compression, during which the stress increases linearly with increasing strain. The second stage is the yield and compaction stage. The stress fluctuates slightly, although the strain increases significantly, indicating that the continuous yield collapse and pore densification of the samples occur at this stage. For the sample with higher porosity, the corresponding strain at this stage is larger because the larger space in the sample requires more compressed deformation from the compact bulk. The third stage is the plasticity of the compacted bulk, which includes work-hardening behavior. Severe plastic deformation occurs after the yield stage. The sample is already compacted and can bear higher stress. The stress continuously increases with increasing strain.

**Figure 7:**
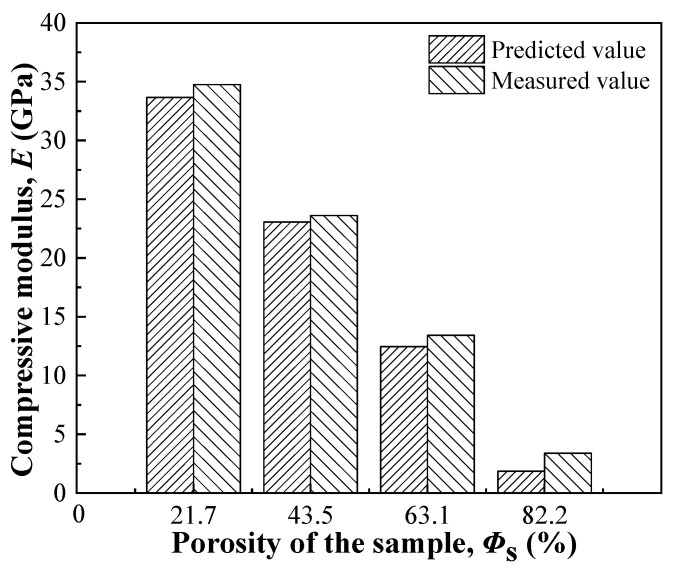
Compressive properties of the prepared samples with different porosities.

Figure 7 shows the compressive moduli of the prepared samples, which were predicted by Equation (9) using *Φ*_s_ instead of *Φ*_m_ and measured by compression tests, respectively. The predicted and measured moduli both decreased with an increase in porosity, and the difference between them was <1.5 GPa, indicating that Equation (9) is suitable for predicting the moduli of irregular prismatic porous structures when they are designed. Therefore, a computational model design can adjust the modulus well using a given irregular prismatic porous structure.

Table 1 lists the mechanical properties of human bone, porous Ta, compact Ti–6Al–4V, and porous Ti–6Al–4V with irregular prismatic porous structures. The compressive modulus and yield strength of the porous Ti–6Al–4V are lower than those of the compact Ti–6Al–4V and decrease with increasing porosity. Compared with compact Ti–6Al–4V, porous Ti–6Al–4V with a porosity of 21.7% shows a nearly 70% decrease in modulus, but the yield strength decreases by only approximately 30%. This suggests that the irregular prismatic porous structure effectively reduces Young’s modulus without a large strength loss. The compressive yield strength of porous Ti–6Al–4V is much higher than that of porous Ta, although they have similar porosities. Furthermore, the elastic modulus of porous Ti–6Al–4V is similar to that of cortical bone, suggesting better biomechanical compatibility. Therefore, the irregular prismatic porous structure designed in this study meets the requirements of medical porous structures and has considerable application potential.

#### 3.3.3. Magnetic Susceptibility

**Figure 8:**
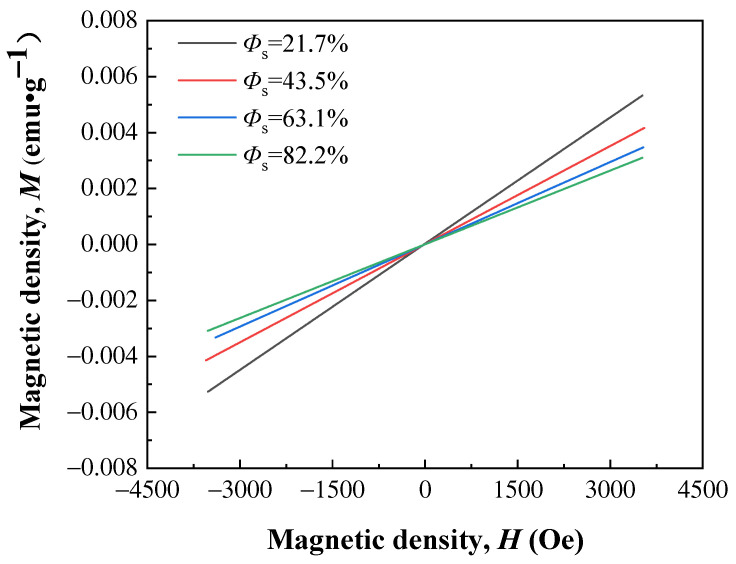
*M*–*H* curves of the porous Ti–6Al–4V with irregular prismatic porous structure.

**Figure 9:**
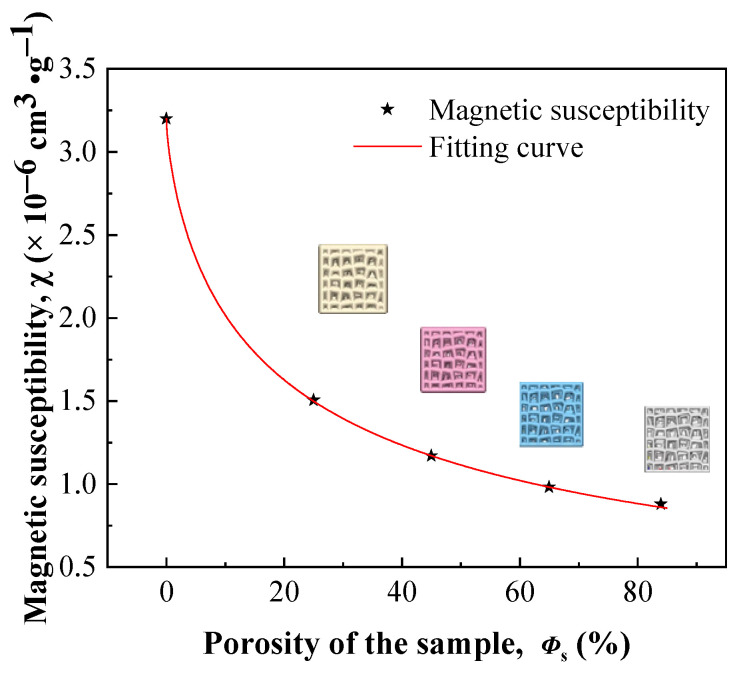
Mass magnetic susceptibility of the porous Ti–6Al–4V with different porosities.

The magnetizations of porous Ti–6Al–4V exhibited a good linear correlation with the applied magnetic field, as shown in Figure 8. The slope of the *M*–*H* curve decreases with an increase in porosity, suggesting a decrease in mass magnetic susceptibility. The mass magnetic susceptibilities of porous Ti–6Al–4V calculated from Figure 8 are presented in Figure 9, together with the mass magnetic susceptibilities of compact Ti–6Al–4V. The relationship between the mass magnetic susceptibility and porosity is fitted using Equation (9), with a correlation coefficient of *R* > 0.99.

(10) $$\chi =-0.175+\frac{3.374}{1+2.51\Phi_{s}^{0.59}}$$

where:

*χ*—Mass magnetic susceptibility (cm^3^∙g^−1^);
*Φ*_s_—Porosity of the sample.

A notable decrease in the magnetic susceptibility was observed for porous Ti–6Al–4V. The magnetic susceptibility of porous Ti–6Al–4V with a porosity of 21.7% was ~1.50 × 10^−6^ cm^3^·g^−1^, which is just ~47% that of compact Ti–6Al–4V (3.20 × 10^−6^ cm^3^·g^−1^) [58]. However, the magnetic susceptibility slightly decreases to 0.88 × 10^−6^ cm^3^·g^−1^, when the porosity is 82.2%. A low magnetic susceptibility and desirable mechanical properties can be obtained by controlling the porosity of porous Ti–6Al–4V with an irregular prismatic porous structure.

## 4. Conclusions

To reduce the Young’s modulus and magnetic susceptibility of Ti–6Al–4V, a mathematical method was used to establish a porous structure by computational modeling, and then the reliability was verified by testing the samples prepared by SLM. Porous Ti–6Al–4V with an irregular prismatic porous structure was computationally modeled using the Voronoi principle. The porosity was mainly determined by the *d*_p_-to-*D* ratio of the prismatic structure and was slightly influenced by the seed number and irregularity. The specific surface area was proportional to the seed number. The average pore size was proportional to the irregularity and inversely proportional to the seed number and *d*_p_-to-*D* ratio. The *d*_p_-to-*D* ratio of the prismatic porous structure had the most significant impact on the pore characteristics and was used as a parameter to adjust the porosity. An equation was derived to calculate the modulus of the porous Ti–6Al–4V designed using the proposed method, and it was suitable for predicting the compressive modulus of the sample subsequently prepared by SLM. Samples with porosities of 21.7%–82.2% had moduli of 3.38–34.75 GPa, which were much lower than that of compact Ti–6Al–4V. The compressive yield strengths ranged from 115 to 574 MPa, which were much higher than those of commercial porous Ta. The mass magnetic susceptibility could be reduced using an irregular prism porous structure. Porous Ti–6Al–4V with a porosity of 21.7% showed a 50% decrease in magnetic susceptibility compared with compact Ti–6Al–4V. The magnetic susceptibility decreased slightly as the porosity increased from 21.7% to 82.2%. The porous Ti–6Al–4V with an irregular prism porous structure showed mechanical properties similar to those of human bone and a much lower magnetic susceptibility than that of compact Ti–6Al–4V. The irregular prismatic porous structure is suitable for designing Ti–6Al–4V with controllable porosity and desirable biomechanical compatibility.

## Figures and Tables

**Table 1:** Mechanical properties of bones, porous Ta, compact Ti–6Al–4V, and irregular prismatic porous Ti–6Al–4V.

Materials	Porosity (%)	Compressive Modulus *E* (GPa)	Compressive Yield Strength *σ*_s_ (MPa)
Spongy bone [55]	50–90	0.02–2	2–12
Cortical bone [56]	3–5	3–30	80–150
Porous Ta [57]	66–88	0.0373–2.2	4–12.7
Compact Ti–6Al–4V [53]	/	~110	~831
Porous Ti–6Al–4V (this study)	21.7%	~34.8	~574
	43.5%	~23.6	~457
	63.1%	~13.4	~345
	82.2%	~3.4	~115

## Data Availability

Data are contained within the article.
